# Efficiency of a non-recycling postharvest fungicide drencher to enhance management of apple decay and food safety

**DOI:** 10.3389/fmicb.2024.1509368

**Published:** 2024-12-19

**Authors:** Achour Amiri, Claire M. Murphy, Gween A. Hoheisel, Clayton L. Haskell, Faith Critzer

**Affiliations:** ^1^Department of Plant Pathology, Tree Fruit Research and Extension Center, Washington State University, Wenatchee, WA, United States; ^2^School of Food Science, Irrigated Agriculture Research and Extension Center, Washington State University, Prosser, WA, United States; ^3^Washington State University Extension, Washington State University, Prosser, WA, United States; ^4^Department of Food Science and Technology, University of Georgia, Athens, GA, United States

**Keywords:** fungicide solutions, non-recycling, application methods, food safety, postharvest diseases

## Abstract

**Introduction:**

Recycling drenchers used to apply postharvest fungicides in pome fruit may spread microorganisms, i.e., plant and foodborne pathogens, that increase fruit loss and impact food safety.

**Methods:**

A non-recycling field drencher (FD), which drenches unstacked bins of fruit, was compared to a commercial recycling packinghouse drencher (CPD) for fruit coverage, fungicide residues, postharvest diseases control and spread of plant pathogens, total coliforms and generic *Escherichia coli*. A mixture of fludioxonil (FDL) and thiabendazole (TBZ) was used in 2021, while pyrimethanil (PYR) was applied in 2022 to alternate fungicides.

**Results:**

The overall spray coverage assessed with pyranine was not significantly different between the FD and CPD. The residue levels of FDL and TBZ were similar between the two methods on Honeycrisp apples at the top, middle, and the bottom of the bins, whereas the residue levels of PYR were significantly lower at the bottom of the bins treated through the FD. The density of plant pathogens and overall disease incidence were similar on apples drenched through both systems in 2021 and significantly lower in FD-treated apples in 2022. The incidence of blue mold, the most important postharvest disease caused by *Penicillium* spp., was significantly lower in apples treated through the FD in both years. The levels of total coliforms and generic *E. coli* were significantly higher in fungicide solutions collected from the CPD compared to the FD. Total coliforms increased significantly on apples treated via the CPD but not on apples treated through the FD.

**Discussion:**

Findings from this study suggest that the new non-recycling drencher has potential as an alternative to recycling packinghouse drenchers in reducing the spread of plant and foodborne pathogens.

## 1 Introduction

In the U.S. Pacific Northwest (PNW), several fungicides from six different chemical groups are registered and applied preharvest to manage pre- and postharvest diseases of pome fruit (Amiri, 2024). The efficacy of these fungicides in reducing postharvest diseases varies based on the target pathogen, timing of application, weather conditions, and fruit maturity. Fruits may be infected in orchards by latent pathogens such as *Alternaria* spp., *Neofabraea* spp., *Phacidiopycnis* spp., and *Botrytis cinerea* (Henriquez et al., 2004; Kim and Xiao, 2006; Amiri and Ali, 2016). Moreover, wounds and bruises sustained to fruit during harvest, transportation, and handling may serve as entry points for *Penicillium* spp. and *Mucor* spp., leading to further infections and fruit loss during storage (Bondoux, 1992; Amiri and Bompeix, 2005a). Conventional packers typically apply single-site fungicides, i.e., thiabendazole, pyrimethanil, fludioxonil or difenoconazole, in addition to the multisite fungicide Captan, to protect fruits during the extended period of cold storage, which can last up to 12 months.

For decades, postharvest fungicides have been applied to apples and pears through drench applications. Upon arrival at the packinghouse, a semi-truck with bins of fruits stacked two to three high is driven through a drencher under which a fungicide solution is applied directly to the fruits within a few hours after harvest. The drenching process is intended to provide enough spray coverage to control diseases, extend postharvest shelf life, and maintain fruit quality. However, the fungicide solution in the drencher is collected and recirculated to treat up to 1,000 bins of fruit. This recirculation may increase the accumulation of dirt, debris, and microorganisms, which may increase the risk of fruit contamination with plant and human pathogens. Additionally, managing the waste from large volumes of fungicide solutions is costly, adding another drawback to conventional packinghouse drenching of fruit. Alternatives to recycling packinghouse drenchers, such as thermonebulization (TNB), also called thermal fogging (Mogia et al., 2003; Vardar et al., 2012), has been used by fruit packers in the U.S. PNW and other growing regions to apply fungicides to pome fruit postharvest. While TNB eliminates the risk of cross-contamination, fungicide residue levels may vary significantly within the room and the bins (Delele et al., 2012). This variation can lead to safety issues related to high maximum residue level (MRLs) and reduced efficacy due to lower residue levels in certain parts of the room (Koundal and Amiri, 2018). Thus, the pome fruit industry has a growing interest in improved fungicide application methods to address these challenges.

The use of recirculated water and fungicide solutions during postharvest handling poses considerable risks for cross-contamination and food safety. During this process, bacteria present in the solution or on produce surfaces may contaminate the water. Bacteria can proliferate in recycled solutions, thereby increasing the risk of contaminating large volumes of produce. Previous research and published risk assessments have demonstrated that without proper treatment and regular monitoring, recirculated water can become a vehicle for transferring pathogens from one batch of produce to another (Danyluk and Schaffner, 2011; Jensen et al., 2015; Gomba et al., 2017; Maffei et al., 2017; De et al., 2018; Mokhtari et al., 2018). While the context is typically not associated with fungicide drenching, the concept is the same and has been previously identified as a risk factor (Pietrysiak et al., 2019). Furthermore, postharvest water has been linked to or suspected to be a contributing factor in numerous produce outbreaks (FDA, 2011, 2019; Waters, 2012). Fungicides incorporated into recirculated drench systems are not intended to control foodborne pathogens. In fact, many are not active against foodborne pathogens and have been shown to support their growth, raising concerns that these systems could serve as a reservoir for cross-contaminating large volumes of fruit with foodborne pathogens (Gomba et al., 2017; Guan et al., 2001). Thus, implementing effective management strategies that minimize the use of recirculated water is crucial for preventing outbreaks and mitigating foodborne illnesses in conformity with regulations of the Food Safety Modernization Act (FSMA), established in 2011, which rendered sanitation of recycling fungicide drenchers mandatory in the USA.

The existing issues associated with fungicide application via recycling packinghouse drenching systems warrant the development and validation of alternative fungicide applications that eliminate the recirculation of water while providing equal or better efficacy for controlling plant pathogens. The few studies that attempted to develop and assess the efficacy of portable or non-recycling drenching systems revealed potential challenges for practical applications and did not assess the impact on food safety (Janisiewicz et al., 2005; Rosenberger et al., 2010). A new single-pass fungicide drencher, which does not recycle the fungicide solution, was developed in Washington state (WA) to apply postharvest fungicides and has been increasingly gaining interest from fruit packers in the region. In this study, research was conducted to compare the novel field drencher (FD) to a commercial packinghouse drencher (CPD) in terms of (i) spray coverage and fungicide residue levels, (ii) efficacy of fungicides for postharvest decay pathogens, and (iii) quantities of total coliforms and *Escherichia coli* in fungicide solutions and on apple surfaces.

## 2 Materials and methods

### 2.1 Field and packinghouse drencher designs

**Figure 1:**
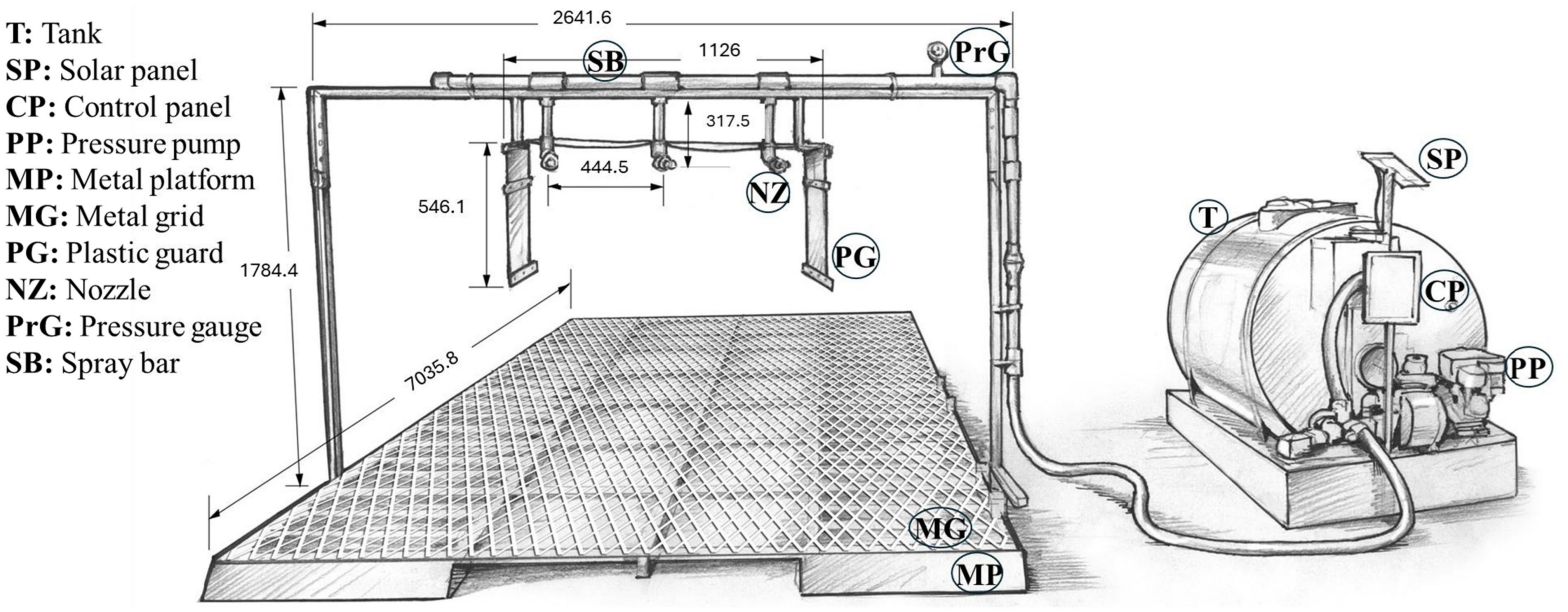
Illustration of the non-recycling field drencher (FD) developed and tested in this study. Dimensions are given in mm.

The field drencher (FD) system consisted of a 1,514 L mixing tank connected to a spray bar equipped with three nozzles (QCTF-VS20 Quick Turbo FloodJet Wide Angle Flat Spray Tip) and plastic guards on the side to reduce drift (Figure 1, Supplementary Figure S1A). The spray system is activated using a key fob as a tractor carrying a bin-trailer with four unstacked bins is driven underneath a 1.12 m spray bar on a metal platform (7.03 m × 1.2 m × 0.1 m) used to collect the solution running-off from the bins. The FD operates at 1 bar, delivering a flow rate of 27.6 L/min through three nozzles applying approximately 5.6 L of solution per bin in 12 s, the average time for a bin to pass beneath the spray bar. The four treated bins were allowed to drain for 30 s and the driver reversed to similarly treat the fruits a second time. The commercial packinghouse drencher (CPD) consisted of a 7,570 L tank connected to a rectangular spray head (2.6 × 3.8 m) delivering approximately 9,500 L/min for about 30 s. In this study, 24 bins were positioned in three high stacks on a semi-truck driving beneath the CPD (Supplementary Figure S1B). The dripping solution was recycled from a port on the ground into the tank and recirculated to treat about 600 bins.

### 2.2 Assessment of spray deposition patterns of the FD and CPD

Apple bins (1.2 m × 1.2 m × 0.64 m) were modified to create four rectangular sections, each approximately 16 cm in height. Two reinforcing bars were inserted at each side of each section to establish a small apple-free zone within each section. To assess the deposition of both FD and CPD drenchers, two pipe cleaners (n = 4 top, 4 bottom per bin) were secured on poles inserted into each of the four apple free zones. Pyranine (Acros Organic, Fair Lawn, NJ) was mixed in the FD and CPD spray tanks at 83 mg/L and 328 mg/L, respectively, considering the volume of water used in each system. Four bins were treated for each drencher type and the experiment was repeated four times. Bins were allowed to drain for 30 min and the pipe cleaner samples were bagged, labeled, and stored at 4°C in the dark for analysis. Tank samples were collected pre- and post-drenching for each of the four replications. Depending on sample concentration, 60 to 180 mL of sterile deionized water was added to each bag. The bags were vigorously shaken for 30 s, allowed to settle, and an aliquot from each bag was analyzed with a 10-AU fluorometer (Turner Designs, San Jose, CA, United States). Data from fluorometry were corrected using a calibration curve generated using standard dilutions for each application. Deposition data was then normalized for treatment comparison using a tank sample ratio and dilution aliquot. Sampling for all the experiments described below were conducted from the same apple lots and fungicide solutions treated or applied via the FD and CPD.

### 2.3 Quantification of the total and fungal microbiota in fungicide solutions and on the surface of apples treated through the FD and CPD

The mixture of fludioxonil (FDL) (Shield-Brite^®^ FDL-230SC, Pace International LLC., Wapato, WA) and thiabendazole (TBZ) (Shield-Brite^®^ TBZ500D, Pace International LLC.) was applied in 2021, whereas pyrimethanil (PYR) (Shield-Brite^®^ Penbotec 400SC, Pace International LLC.) was applied in 2022 in accordance with recommendations for fungicide alternation. Mixing TBZ and FDL is common to optimize FDL efficacy. Peracetic acid (PAA, Shield-Brite^®^ PAA 5.6; 5.6% peroxyacetic acid and 26.5% hydrogen peroxide) was added to both tanks at 7.8 mL/L. In 2021, fungicide solutions running-off from the bins treated through the FD were aseptically collected during drenching of Honeycrisp apples harvested at commercial maturity from four different lots (Hc902, Hc1139, Hc1156, and Hc1918). Six fungicide samples were collected from each lot at different points during drenching. For the CPD, eight fungicide samples, i.e., two samples collected at each sampling point were collected in labeled sterile 50 mL tubes from two separate replicate tanks as 0, 100, 200, and 600 bins had been treated with the same fungicide solution. To assess the culturable microbiota on the apples, 16 bins from the same Honeycrisp lots were set in a randomized complete block design of four replicate bins each. Four apples per bin were sampled from each lot before the fungicide was applied and used as a control, and four apples per bin were sampled 30 min after the fungicide was applied. For the CPD, apples from the same lots were similarly collected after 200 bins had been treated with the fungicide solution of the same tank. Fungicide and apple samples were transported in coolers to the laboratory, stored at 4°C for ≤48 h before enumeration. Apples were individually immersed in 100 mL of buffered peptone (Becton, Sparks, MD) water with 0.1% Tween 80 (G Biosciences, St. Louis, MO) and placed on a rotary shaker at 160 rpm for 30 min. To enumerate the colonies in the fungicides and on the apples, 100 mL aliquots from each sample were spread onto 9-cm Petri dishes containing 1% MEAT malt extract agar (Amiri and Bompeix, 2005b) amended with 0.05% of Triton X100 (Amresco LLC., Solon, OH). Three MEAT plates/sample/lot were used. The plates were incubated for 7 days at 20°C, colonies were enumerated, and the fungal colonies were identified to the genus level (Dugan, 2006). Spore and cell concentrations in the solutions were expressed as colony-forming unit (CFU)/mL or CFU/cm^2^ of the apple surface. The experiment was repeated in 2022 using the same procedure and fruit lots used in 2021.

### 2.4 Fungicide residue levels in tank solutions and on apples treated through the FD and CPD

Residue levels of FDL and TBZ were assessed in 2021 and residues of PYR were analyzed in 2022. Thirty apples were collected from each experimental bin (four bins per lot) from the four Honeycrisp lots mentioned above. For the FD, bins one to four on the trailer were sampled (Supplementary Figure S1A). For the CPD, apples were collected from two bins from the 3^rd^ (Top), 2^nd^ (Middle), and 1^st^ (Bottom) high bins on the stacks for each lot (Supplementary Figure S1B). Each bin was divided into three sections, top, middle, and bottom of 21 cm each, in a way to sample 10 apples from each section using a similar pattern, i.e., two apples at each corner and two at the center of the section. The 10 apples from each section and bin were placed in a clean labelled mesh bag, transported to the laboratory, stored at 1.5°C, and fungicide residues were analyzed within 15 days. The apples were sliced into quarters and one quarter from each apple was mixed with nine others from the remaining apples of each sample to make one composite sample. Three samples were analyzed from each bin section and lot. The samples were weighted, blended separately in a solvent, and resulting samples were centrifuged at 8,000 rpm for 15 min. Aliquots of the supernatant were anonymously coded and analyzed using a Hewlett Packard 6,890 N gas chromatograph with an NPD detector at Pace International LLC (Wapato, WA) using proprietary methods. The fungicide residue levels on apples were expressed as mg of the active ingredient per kilogram of apple (mg/kg). The concentration of TBZ, FDL, and PYR in the fungicide solutions applied through the FD and CPD was analyzed in the same samples collected for microbiota enumeration experiment described above.

### 2.5 Efficacy of postharvest fungicides applied through the FD and CPD to control postharvest diseases in long term cold storage

In September of 2021, Honeycrisp apples, from the same aforementioned four lots were harvested at commercial maturity and treated on the same day. For the FD, 16 replicate bins from each lot, set in four replicates of four bins each, were treated with a mixture of formulated FDL and TBZ at the label rates of 1.25 mL/L and 1.25 mL/L, respectively, at the vicinity of the orchard near Sellah, WA. For the CPD, bins from the four lots were transported, in three bins per stack (Supplementary Figure S1), on a semi-truck and drenched at the packinghouse facility, Sellah, WA, with a fungicide solution that had already been used to treat 200 commercial bins. For both treatments, 100 apples were collected from the top 40 cm zone of each bin before the fungicides were applied and four other samples were collected from the same bins 30 min post-drench. The 100 apples from each sample were stored in separate labeled crates in a regular atmosphere (RA) at 10°C for 10 days then at 2.7°C for up to 8 months. Apples were inspected every two months for decay incidence and decay types. In September of 2022, the experiment was repeated with the same apple lots but were treated with PYR at 2.5 mL/L following the same experimental design and sampling procedure used in 2021.

Additionally in 2022, eight commercial bins per lot, i.e., bins from the four Honeycrisp lots mentioned above, and eight bins from three Gala apple lots (Ga901, Ga1113, and Ga1124) treated through FD or CPD, were stored at a commercial facility and investigated. The two cultivars were planted in nearby orchards and are expected to be under similar weather conditions. None of the cultivars received a preharvest fungicide application. The sets of eight bins each were treated through the FD or CPD on the same day and tanks used for the aforementioned trial, labeled, and stored at 1.5°C for Gala and 2.7°C for Honeycrisp in a controlled atmosphere (CA, 1.2% O_2_ and 0.5% CO_2_) at a commercial cold storage facility in Selah, WA. After 10 months of storage, all fruit in the bins were inspected manually. Asymptomatic and symptomatic apples were counted to calculate disease incidence in each bin and lot. The decayed apples were collected in clamshells and transported to the laboratory for decay identification.

### 2.6 Quantification of total coliforms and *E. coli* in fungicide solutions and on apples treated through the FD and CPD

To quantify total coliforms and generic *E. coli* levels in the fungicide solutions applied through the FD and CPD, four samples were collected from eight separate tanks for a total of 32 samples per drencher type. The samples (100 mL) were aseptically captured into sterile bottles, transported on ice to the laboratory, and processed within 6 h. Total coliform and generic *E. coli* levels were enumerated using Colilert Quanti-Tray 2000 kit (IDEXX, Westbrook, ME) following the manufacturer’s instructions. After incubation for 24 h at 35°C, Quanti-Trays were observed for change in color from colorless to yellow (total coliform) and the presence of fluorescence (*E. coli*) among wells to determine the Most Probable Number (MPN)/100 mL for target cell populations.

To quantify total coliforms and *E. coli* on apples, 12 individual apples were collected from eight tank mixes as described above before and after FD and CPD treatment per replicate, in a completely randomized block design. Apples were each placed in a sterile stomacher bag (VWR, Radnor, PA, United States) and held at 4°C until processed. A 100-mL volume of buffered peptone water with 0.1% Tween 80 (G Biosciences, St. Louis, MO) was added to each bag and rubbed by hand for 30 s to suspend bacteria in the buffer. Samples were serially diluted in 0.1% peptone (Becton, Sparks, MD) and plated in duplicate on total coliform/*E. coli* Petri film (3M, Saint Paul, MN). The films were incubated for 24 h at 35°C after which colonies were enumerated according to manufacturer’s instructions.

### 2.7 Data analysis

To assess spray coverage, a linear mixed effects model was used to characterize the tracer concentration (ng/cm^2^) by zone and location. We assessed model assumptions by examining residual plots, a Shapiro–Wilk test for normality and a Breusch-Pagan test for variance homogeneity. We found some deviations from both assumptions; however, linear mixed effects regression models are robust to small deviations normality for sufficiently large sample sizes. Regarding variance homogeneity, our assumptions may be limited by moderate violations, however, we chose to retain the linear mixed effects regression to maintain simplicity and interpretability. Zone, location, and their interaction were included in the model as fixed effects, while the replicate was included as a random effect. An ANOVA assessed the effects of zone, location and the interaction with Kenward-Rodger degrees of freedom. Least squares means and 95% confidence intervals were extracted for each zone × location combination. Pairwise differences with 95% confidence intervals were extracted between the top and bottom for each zone, with no family-wise adjustment for multiplicity. The total disease incidence and fungicide residue level data were analyzed each year separately. Data were not normally distributed, therefore, the Kruskal-Wallis followed by Dunn’s test with Benjamini-Hochberg *p*-value adjustment was used (*p* = 0.05). Populations of total fungal microbiota, total coliforms and *E. coli* were log-transformed and results were checked for normality and homogeneity of variance. The averages and standard deviations of log CFU/apple or log MPN/100 mL of fungicide solution for fungal microbiota, total coliforms and generic *E. coli*, categorized by FD and CPD, were computed, and significant differences were assessed using Tukey’s honest significance difference test. All analyses were performed in R.

## 3 Results

### 3.1 Spray deposition patterns of the field and commercial packinghouse drenchers

**Figure 2:**
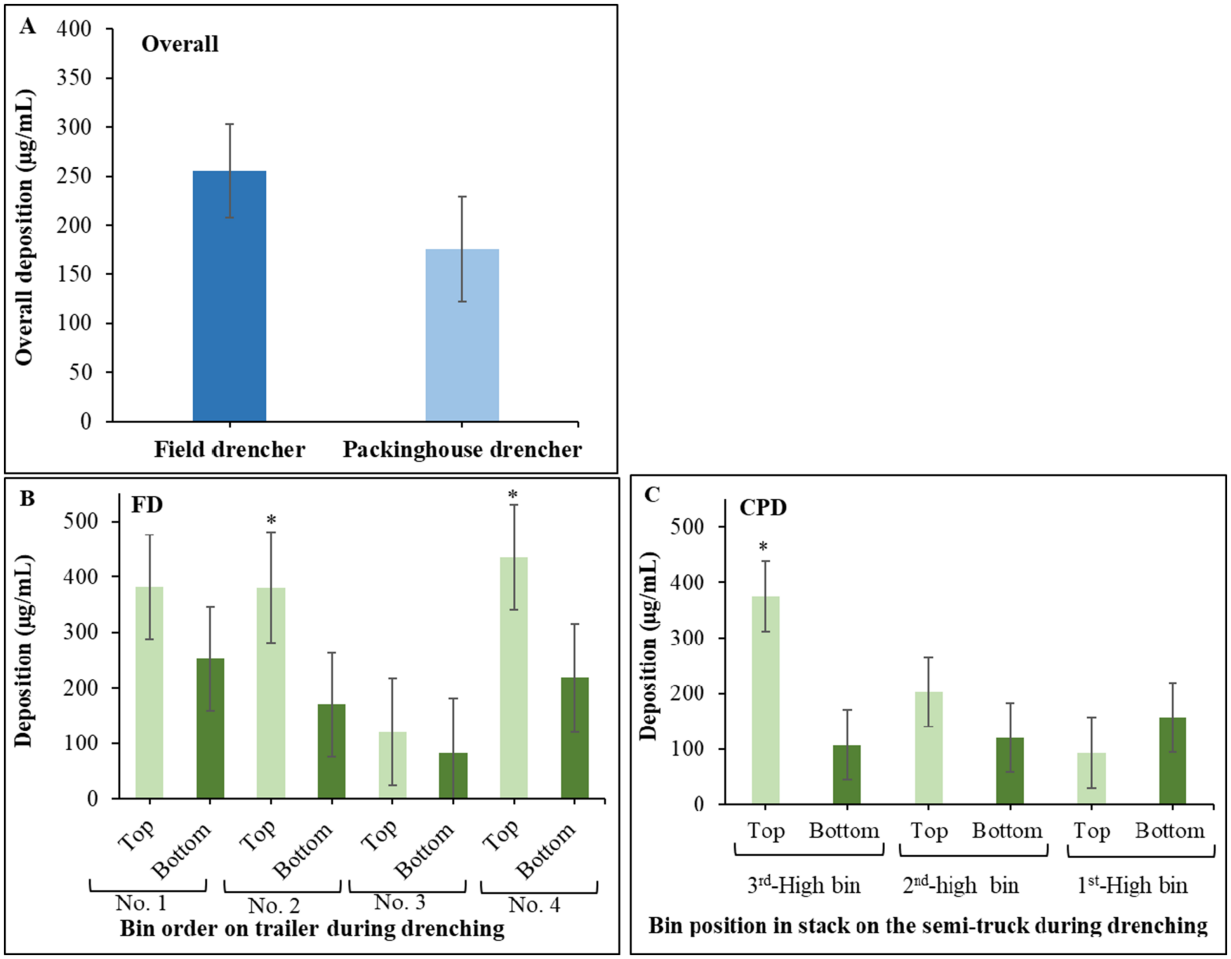
**(A)** Overall pyranine deposition in samples collected from bins drenched through the field drencher (FD) and the commercial packinghouse drencher (CPD). Data bars are the mean and standard errors of 32 samples per drencher type. **(B)** Deposition of pyranine in samples collected from the top and bottom sections of the bins drenched through the FD. **(C)** Deposition of pyranine in samples collected from the top and bottom sections of the bins at three different positions 1st, 2nd and 3rd high on the truck during drenching through the CPD. Data bars are the mean and standard errors of 36 values from nine samples per bin section and four bins per drencher type. An asterisk indicates significant differences based on Tukey’s test at *p* ≤ 0.05.

The overall deposition was higher in the non-recycling FD (255.4 μg/mL) but not significantly different (*p* = 0.27) from the recycling CPD (175.9 μg/mL) (Figure 2A). In the non-stacked bins drenched through the FD, deposition was higher on the top versus bottom sections of two out of four bins (Figure 2B). In the stacked bins treated through the CPD, deposition was significantly (*p* < 0.001) higher on the top bin section of the 3^rd^ high bin on the truck, whereas deposition was overall uniform among the other zones and stacked bins (Figure 2C).

### 3.2 Fungicide residue levels on apples and in tank solutions applied through the FD and CPD

**Figure 3:**
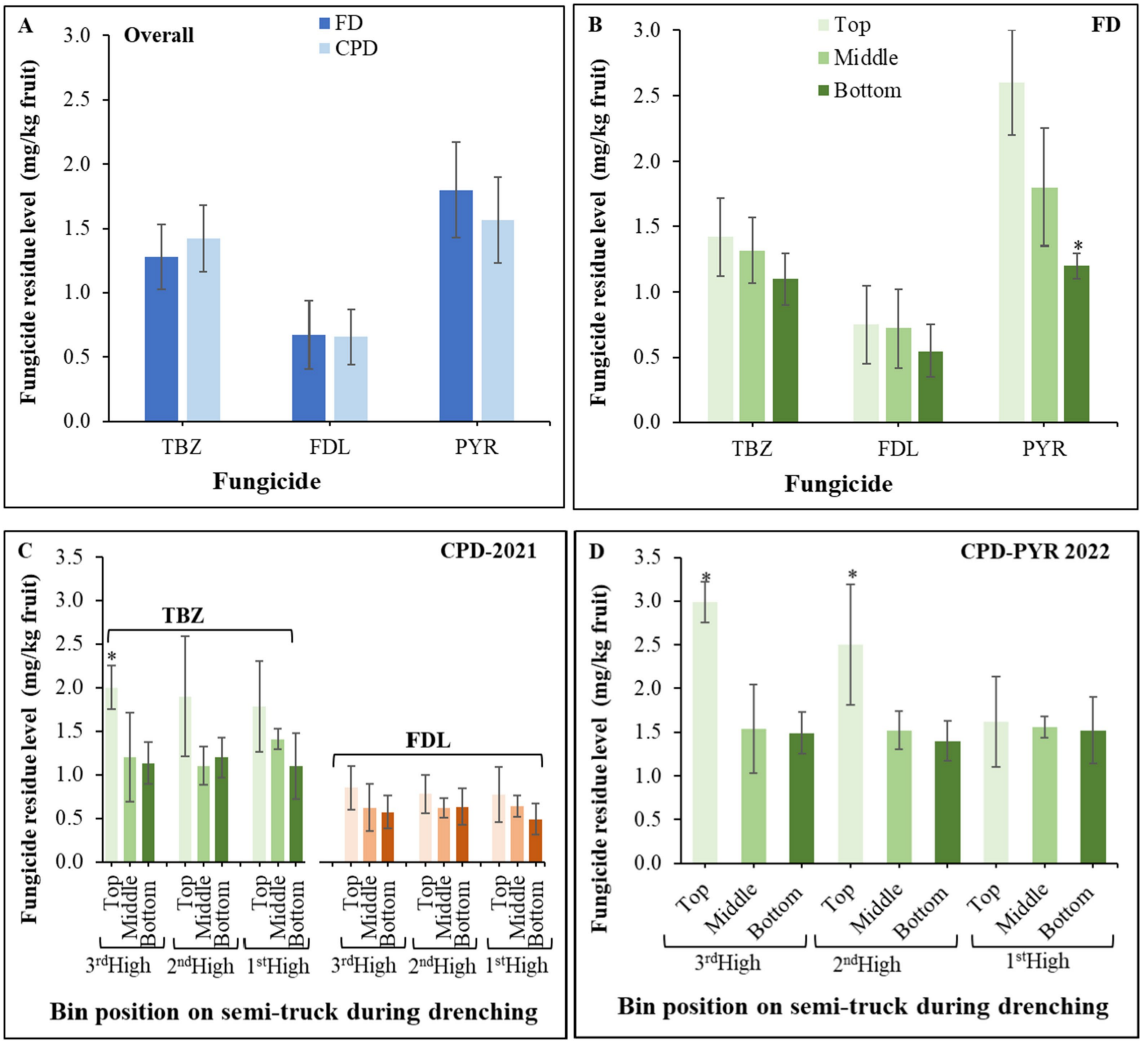
Overall residue levels of thiabendazole fludioxonil and pyrimethanil **(A)** on Honeycrisp apples and residue levels on apples collected from the top, middle, and bottom sections of bins treated through the field drencher **(B)** or commercial packinghouse drencher in 2021 **(C)** and 2022 **(D)**, respectively. Data bars are the mean and standard deviations of 16 samples consisting of three samples of 10 fruit-each per bin section, across the four apple lots. An asterisk indicates significant difference based on Tukey’s test at *p* ≤ 0.05.

The overall residue levels of TBZ, FDL and PYR on Honeycrisp apples were not significantly different between the FD and CPD (Figure 3A). There were no significant differences between the four bins drenched through the FD across lots, therefore, the residue level values were averaged. In 2021, the residue levels of TBZ and FDL on Honeycrisp apples were not significantly different (*p* = 0.66) between the top, middle, and bottom sections of the bins regardless of the application method (Figure 3B). In 2022, the overall residue levels of PYR were significantly higher (*p* = 0.04) at the top bin section compared to the middle and top sections of the bins treated via the FD (Figure 3B). Similar to the spray deposition patterns observed with the CPD, residue levels were significantly higher at the top of the 3^rd^ high bin in the stack on the semi-truck for TBZ (Figure 3C) and the top of the 3^rd^ and 2^nd^ high bins for PYR (Figure 3D), but not for FDL (Figure 3C).

**Figure 4:**
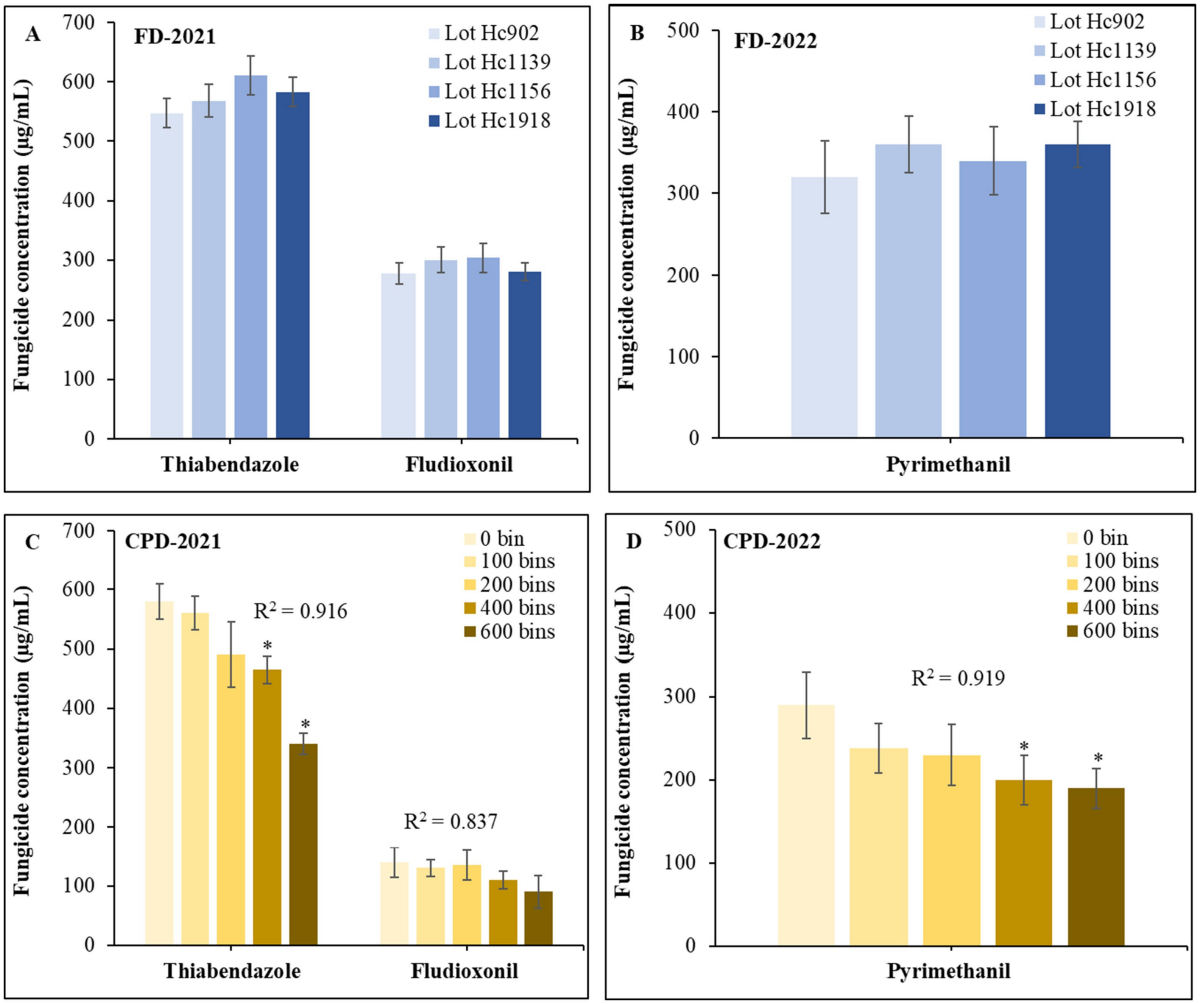
Residue levels of thiabendazole and fludioxonil **(A)** and pyrimethanil **(B)** in fungicide solutions applied through the field drencher (FD); residue levels of TBZ and FDL **(C)** and PYR **(D)** applied through the commercial packinghouse drencher (CPD) after several (0–600) bins had been drenched in 2021 and 2022, respectively. Data bars are the mean and standard deviations of 12 samples consisting of four samples per lot plated to three replicates each for the field drencher or per sampling point (number of bins) for the packinghouse drencher. An asterisk indicates significant difference based on Tukey’s test at *p* ≤ 0.05.

The concentrations of TBZ and FDL in 2021 and PYR in 2022 in the solutions of the FD tanks was similar between lots and ranged from 547 to 610 μg/mL for TBZ, 277 to 303 μg/mL for FDL, and 320 to 360 μg/mL for PYR (Figures 4A,B). The concentrations of the three fungicides in the CPD tanks decreased gradually as more bins were drenched resulting in a positive correlation between the number bins treated with the recycled solution and the concentration of TBZ (R^2^ = 0.92), FDL (R^2^ = 0.84), and PYR (R^2^ = 0.92) in 2021 (Figure 4C) and 2022 (Figure 4D).

### 3.3 Total and fungal microbiota on apples and in fungicide solutions applied through the FD and CPD

In 2021, the mixture of FDL and TBZ applied through the FD and CPD significantly (*p* = 0.03) reduced the total microbiota in four and three Honeycrisp lots, respectively, compared to the control (Table 1). The most frequent fungal pathogen was *Alternaria* spp. which was equally recovered regardless of the application method. The number of propagules of *Penicillium* spp. recovered from Honeycrisp apples at harvest ranged from 0.0 to 0.05 CFU/cm^2^ and was similar between the FD and CPD. In 2022, the total microbiota was significantly lower on apples treated with PYR via the FD compared to the CPD (four lots) and the control (three lots). The density of *Alternaria* spp. was higher on treated compared to the untreated apples and increased in three lots drenched through the FD. The density of *Penicillium* spp. was significantly higher on apples drenched via the CPD in three out of four Honeycrisp lots in 2022 (Table 1).

**Table 1:** Total microbiota and density of *Alternaria* and *Penicillium* spp. on apples treated through the field and packinghouse drenchers.

Orchard	Application method	2021^1^			2022		
Lot #		Total^3^	*Alternaria* spp.	*Penicillium* spp.	Total	*Alternaria* spp.	*Penicillium* spp.
Hc902	Control	3.22 ± 0.5 a	0.35 ± 0.4 a	0.00 ± 0.0 a	72.50 ± 18.2 a	5.40 ± 0.8 de	2.00 ± 0.5 c
	CPD^2^	2.14 ± 0.3 b	0.13 ± 0.2 a	0.00 ± 0.0 a	59.84 ± 8. 9 b	9.30 ± 1.5 d	2.20 ± 0.8 c
	FD^2^	1.48 ± 0.9 b	0.11 ± 0.5 a	0.00 ± 0.0 a	46.94 ± 3.7 c	12.10 ± 2.3 c	2.00 ± 0.7 c
Hc1139	Control	4.19 ± 0.7 a	0.06 ± 0.8 a	0.00 ± 0.0 a	30.87 ± 5.1 d	17.20 ± 5.3 bc	0.40 ± 0.2 d
	CPD	1.96 ± 0.7 b	0.01 ± 0.3 a	0.00 ± 0.0 a	59.84 ± 4.3 b	31.80 ± 6.1 ab	4.40 ± 0.7 b
	FD	2.74 ± 0.8 b	0.55 ± 0.5 a	0.03 ± 0.4 a	37.70 ± 7.1 d	14.80 ± 2.5 c	3.00 ± 0.3 c
Hc1156	Control	3.24 ± 0.5 a	0.20 ± 0.2 a	0.00 ± 0.0 a	71.45 ± 12.8 a	02.40 ± 0.9 f	0.40 ± 0.2 d
	CPD	2.57 ± 0.4 a	0.15 ± 0.3 a	0.00 ± 0.0 a	51.24 ± 11.7 b	04.40 ± 1.3 e	3.30 ± 0.5 b
	FD	0.50 ± 0.1 b	0.04 ± 0.2 a	0.00 ± 0.0 a	38.11 ± 6.6 d	06.00 ± 1.7 de	0.00 ± 0.0 e
Hc1918	Control	5.20 ± 1.9 a	1.10 ± 1.3 a	0.05 ± 0.2 a	62.47 ± 8.7 a	27.70 ± 5.5 b	0.80 ± 0.1 b
	CPD	2.82 ± 0.6 b	0.26 ± 0.5 a	0.00 ± 0.0 a	50.75 ± 9.1 b	13.00 ± 1.9 c	12.0 ± 0.5 a
	FD	2.00 ± 0.4 b	0.83 ± 0.7 a	0.00 ± 0.0 a	40.80 ± 5.1 c	42.00 ± 6.5 a	0.00 ± 0.0 e

1 The mixture thiabendazole + fludioxonil was used in 2021, while pyrimethanil was used in 2022.

2 CPD and FD indicate commercial packinghouse drencher and field drencher, respectively.

3 Total microbiota is expressed as CFU/cm^2^ of the apple surface. Data are the average of 36 values from 12 apples per bin and three replicate plates per fruit for each treatment. Control apples were collected before drenching. Values within the same column followed by different letters are significantly different by Tukey’s test at *p* ≤ 0.05.

*Penicillium* spp. was not isolated from the fungicide solutions of FDL + TBZ applied through the FD or the CPD in 2021, whereas other fungi, i.e., *Alternaria* spp. and *Mucor* spp. were detected at ≤1 CFU/mL regardless of the drencher type (Table 2). In 2022, the density of *Penicillium* spp. ranged from 0.5 CFU/mL in Hc1918 lot to 16.8 CFU/mL in Hc1156 in the PYR solutions applied through the FD (Table 2). The population of other fungi, i.e., *Alternaria* spp. and *Mucor* spp., ranged from 4.8 to 23.3 CFU/mL among the four Honeycrisp lots. The fungal population in the PYR solution drenched through the CPD in 2022 was positively correlated with the number of bins drenched through and increased up to 60.8 CFU/mL for *Penicillium* spp. (R^2^ = 0.94) and 40.2 CFU/mL for other fungi (R^2^ = 0.62), after 600 bins had been drenched (Table 2).

**Table 2:** Density of *Penicillium* spp. and other fungi in fungicide solutions applied through the field and packinghouse drenchers.

Application method	Orchard lot or n of bins drenched^1^	2021		2022	
		*Penicillium* spp.	Other fungi^4^	*Penicillium* spp.	Other fungi
FD	Hc902	0.0 ± 0.0 a^3^	1.0 ± 2.5 a	3.5 ± 5.6 c	23.3 ± 43.5 a
	Hc1139	0.0 ± 0.0 a	0.0 ± 0.0 a	4.7 ± 3.2 c	4.8 ± 12.4 a
	Hc1156	0.0 ± 0.0 a	1.0 ± 3.3 a	16.7 ± 14.4 b	18.8 ± 48.8 a
	Hc1918	0.0 ± 0.0 a	1.0 ± 2.4 a	0.5 ± 1.0 c	19.3 ± 20.0 a
CPD	0^2^	0.0 ± 0.0 a	0.0 ± 0.0 a	0.0 ± 0.0 c	0.0 ± 0.0 b
	100	0.0 ± 0.0 a	1.0 ± 2.0 a	0.0 ± 0.0 c	3.8 ± 4.0 b
	200	0.0 ± 0.0 a	0.0 ± 0.0 a	8.8 ± 4.2 bc	18.8 ± 4.3 a
	400	0.0 ± 0.0 a	0.0 ± 0.0 a	25.4 ± 21.3 b	15.4 ± 21.4 a
	600	0.0 ± 0.0 a	0.0 ± 0.0 a	60.8 ± 14.4 a	40.2 ± 14.5 a

1 The mixture thiabendazole + fludioxonil was used in 2021, while pyrimethanil was used in 2022. The fungicide solutions applied through the field drencher (FD) were collected while fruits from each lot were drenched separately, whereas the fungicide solutions applied through the commercial packinghouse drencher (CPD) were sampled after 0, 100, 200, 400, and 600 bins had been drenched.

2 Number of bins drenched through the CPD.

3 The density is expressed as CFU/mL of the fungicide solution. Data are the average of 6 values from two fungicide samples and three replicate plates per sample for each treatment and lot. Values within the same column followed by different letters were significantly different by Tukey’s test at *p* ≤ 0.05.

4 Other fungi included *Alternaria* spp. and *Mucor* spp.

### 3.4 Efficacy of postharvest fungicides applied through the FD and CPD to control postharvest diseases in long term storage

In 2021, the overall disease incidence was significantly reduced in four and three Honeycrisp lots by FDL + TBZ applied through the FD and CPD, respectively, compared to the untreated control after 8 months of storage at 2.7°C in RA (Table 3). The overall disease incidence was similar between FD and CPD in two lots, Hc902, Hc1139, and significantly lower (*p* = 0.001) when FDL + TBZ were applied through the FD in the Hc1156 and Hc1918 lots (Table 3). Blue mold and gray mold caused by *Penicillium* spp. and *Botrytis* spp., respectively, were the two most frequent diseases found in 2021. Blue mold was significantly reduced (*p* = 0.001) by the FD compared to the CPD in three lots of out of four, whereas the incidence of gray mold was significantly reduced by the FD compared to the CPD in lot Hc902, but the CPD was more effective (*p* = 0.03) in the Hc1139 and Hc1156 lots (Table 3). Speck rot, Mucor rot, bull’s eye rot, and Alternaria rot, caused by *Phacidiopycnis washingtonensis*, *Mucor* spp., *Neofabraea* spp. and *Alternaria* spp., respectively, were found at 1 to 4% and were not impacted by the application method (data not shown).

**Table 3:** Overall and specific disease incidence of blue mold and gray mold on Honeycrisp apples treated with postharvest fungicides through the field and packinghouse drenchers and stored in a regular atmosphere.

	Application method	2021			2022		
Lot #		Total incidence (%)^1^	Blue mold	Gray mold	Total incidence (%)	Blue mold	Gray mold
Hc902	Control	67.0 ± 10.2 a^2^	3.0 ± 1.2 c	63.0 ± 8.7 a	23.0 ± 7.2 ab	23.0 ± 7.2 a	0.0 ± 0.0 e
	CPD	12.0 ± 2.5 b	10.0 ± 2.1 b	2.0 ± 0.7 c	5.0 ± 0.9 d	5.0 ± 0.9 b	0.0 ± 0.0 e
	FD	13.0 ± 2.1 b	13.0 ± 2.9 b	0.0 ± 0.0 d	0.0 ± 0.0 g	0.0 ± 0.0 f	0.0 ± 0.0 e
Hc1139	Control	15.0 ± 1.5 b	11.0 ± 2.7 b	0.0 ± 0.0 d	12.0 ± 2.1 c	8.0 ± 2.4 b	3.0 ± 1.2 c
	CPD	13.0 ± 2.6 b	13.0 ± 1.9 b	0.0 ± 0.0 d	5.0 ± 1.5 d	3.0 ± 0.9 c	1.0 ± 0.2 d
	FD	6.0 ± 1.3 c	9.0 ± 1.1 c	2.0 ± 0.9 c	0.0 ± 0.0 g	0.0 ± 0.0 f	0.0 ± 0.0 e
Hc1156	Control	8.0 ± 2.2 c	8.0 ± 2.4 c	0.0 ± 0.0 d	32.0 ± 9.1 a	3.0 ± 1.1 c	29.0 ± 5.6 a
	CPD	3.0 ± 0.7 d	3.0 ± 0.9 d	0.0 ± 0.0 d	3.0 ± 0.9 de	3.0 ± 0.8 c	0.0 ± 0.0 e
	FD	2.0 ± 1.0 d	0.0 ± 0.0 e	1.0 ± 2.2 c	1.0 ± 0.5 f	1.0 ± 0.7 d	0.0 ± 0.0 e
Hc1918	Control	48.0 ± 12.1 a	24.0 ± 4.8 a	21.0 ± 3.8 b	20.0 ± 6.1 ab	5.0 ± 1.7 b	9.0 ± 2.0 b
	CPD	14.0 ± 2.4 b	15.0 ± 5.5 b	2.0 ± 0.8 c	3.0 ± 1.5 de	3.0 ± 0.8 c	0.0 ± 0.0 e
	FD	7.0 ± 1.7 c	5.0 ± 1.7 c	2.0 ± 0.9 c	5.0 ± 0.9 d	2.0 ± 0.5 c	2.0 ± 2.7 e

1 Decay incidence (%) ± standard deviations of means from the four replicates after 8 months of storage at 2.7°C in a regular atmosphere. Apples were treated with a mixture of thiabendazole and fludioxonil in 2021 and with pyrimethanil in 2022 through the field drencher (FD) and the commercial packinghouse drencher (CPD).

2 Values of the overall disease incidence followed by different letters were significantly different by Dunn’s test at *p* ≤ 0.05.

In 2022, PYR applied through the FD reduced the overall disease incidence significantly (*p* = 0.02) in three lots, Hc902, Hc1139, and Hc1156, compared to the CPD, and both drenchers reduced the diseases incidence significantly compared to the control (Table 3). Like in 2021, the incidence of blue mold was significantly reduced in the FD-treated apples compared to the CPD in three Honeycrisp lots, whereas the incidence of gray mold was significantly reduced by the FD in lot 1,139 and equal between the two application methods in the other three lots (Table 3).

In the large trial using full bins treated with PYR in 2022 and stored under CA in the commercial cold facility, the overall disease incidence after 10 months of storage was similar between the FD and CPD in seven lots and was significantly lower in Gala lot Ga1124 treated through the FD (Table 4). Six major postharvest diseases, i.e., blue mold, gray mold, Alternaria rot, speck rot, bull’s eye rot and Mucor rot, were detected with blue and gray molds making for up to 70% of total diseases. The incidence of blue mold and Mucor rot was significantly reduced (*p* = 0.002) in six out of seven lots when PYR was applied through the FD compared to CPD (Table 4). On the other hand, the incidence of gray mold and bull’s eye rot were significantly reduced by the CPD in four lots compared to FD.

**Table 4:** Overall and specific disease incidence of major postharvest diseases in Honeycrisp and Gala apple drenched with pyrimethanil through the field and packinghouse drenchers in 2022 and stored in a commercial controlled atmosphere.

Cultivar			Overall disease incidence and incidence of major diseases^1^						
	Lot #	Method	Overall	Blue mold	Gray mold	Alternaria rot	Speck rot	Bull’s eye rot	Mucor rot
Honeycrisp	Hc902	CPD	1.8 ± 0.5 b	53.3 ± 3.5 b	36.2 ± 7.8 b	1.3 ± 0.3 c	2.6 ± 1.1 b	6.6 ± 1.5 b	0.0 ± 0.0 e
		FD	1.6 ± 0.2 b	37.5 ± 4.6 c	46.9 ± 9.5 ab	6.3 ± 1.5 a	4.7 ± 1.5 b	4.7 ± 1.1 bc	0.0 ± 0.0 e
	Hc1139	CPD	3.3 ± 0.4 b	64.7 ± 9.3 a	17.9 ± 3.8 c	2.9 ± 0.4 bc	5.4 ± 3.5 ab	2.9 ± 0.9 c	5.0 ± 1.5 c
		FD	3.2 ± 0.4 b	43.7 ± 7.5 b	13.8 ± 3.5 c	1.6 ± 0.5 bc	10.9 ± 4.5 ab	12.8 ± 2.9 a	15.7 ± 8.0 a
	Hc1156	CPD	2.5 ± 0.3 b	55.1 ± 8.5 b	20.7 ± 4.6 c	1.8 ± 0.9 bc	1.8 ± 0.7 b	2.6 ± 0.9 c	17.1 ± 3.2 a
		FD	2.5 ± 0.7 b	42.9 ± 9.1 b	38.3 ± 6.6 b	3.7 ± 1.9 ab	5.0 ± 1.5 b	5.0 ± 1.5 b	5.0 ± 1.7 c
	Hc1918	CPD	15.0 ± 5.4 a	52.0 ± 5.9 b	9.8 ± 1.3 d	0.8 ± 0.5 c	2.4 ± 1.9 b	12.7 ± 2.7 a	21.5 ± 3.4 a
		FD	7.1 ± 5.4 ab	36.3 ± 3.3 c	17.2 ± 3.1 c	2.1 ± 1.1 b	14.1 ± 6.5 a	19.3 ± 4.5 a	11.0 ± 2.3 b
Gala	Ga901	CPD	0.2 ± 0.1 e	51.6 ± 6.5 b	12.9 ± 3.7 c	6.5 ± 1.8 a	3.2 ± 2.1 b	6.5 ± 1.3 b	19.4 ± 2.5 a
		FD	0.3 ± 0.2 de	35.7 ± 4.4 c	40.4 ± 6.1 b	4.8 ± 1.1 a	7.1 ± 2.5 ab	4.8 ± 1.4 b	7.1 ± 3.5 bc
	Ga1113	CPD	0.6 ± 0.2 cd	27.9 ± 8.8 c	36.0 ± 5.5 b	4.7 ± 0.6 a	3.5 ± 1.1 b	2.3 ± 0.8 c	25.6 ± 5.7 a
		FD	0.9 ± 0.1 c	30.4 ± 5.1 c	58.2 ± 8.8 a	1.7 ± 0.4 b	2.6 ± 0.8 b	4.3 ± 1.1 b	2.6 ± 0.9 d
	Ga1124	CPD	2.1 ± 0.3 b	63.3 ± 3.9 a	11.6 ± 2.2 c	1.6 ± 0.5 b	1.8 ± 1.5 b	2.8 ± 0.9 c	18.5 ± 3.8 a
		FD	0.4 ± 0.2 cd	55.8 ± 3.5 b	15.1 ± 3.9 c	0.0 ± 0.0 d	4.7 ± 2.5 b	6.9 ± 1.9 b	12.8 ± 2.2 b

1 Decay incidence (%) ± standard deviations of means from the four replicates after 10 months of storage in commercial storage rooms in 1.2% O_2_ and 0.6% CO_2_ at 1 and 2.7°C for Gala and Honeycrisp, respectively. Incidence is expressed as the total decayed fruit related to healthy fruit counted in eight bins per lot. Values within the same column followed by different letters were significantly different by Dunn’s test at *p* ≤ 0.05.

### 3.5 Levels of total coliforms and generic *E. coli* on apples and in fungicide solutions applied through the FD and CPD

The levels of total coliforms (log MPN/100 mL) were significantly higher (*p* ≤ 0.05) in fungicide solution samples collected from the CPD (5.71 log MPN/100 mL) compared to those from the FD (3.59 log MPN/100 mL) (Table 5). A significant difference (*p* ≤ 0.05) was also observed for generic *E. coli* levels, with 3.03 and 1.26 log MPN/100 mL in the CPD and FD, respectively (Table 5). The levels of total coliforms as well as *E. coli* on the apple surfaces before and after the FD fungicide application were not significantly different and remained at or below the limit of detection before (2.00 log CFU/apple) and after (2.08 log CFU/apple) drenching through the FD (Table 5). However, total coliform levels on apples treated through the CPD increased significantly (*p* ≤ 0.05) from 2.02 log CFU/apple before drenching to 3.96 log CFU/apple after drenching (Table 5).

**Table 5:** Average concentrations of total coliforms and generic *Escherichia coli* in the fungicide solutions of the field and packinghouse drenchers and on apple surfaces before and after drenching.

	Method of application		Indicator Organism^1^	
Type of sample		Sampling point	Total coliforms^3^	Generic *E. coli*^4^
Fungicide solutions	CPD^2^	During treatment	5.71 ± 0.51 c	3.03 ± 1.36 b
	FD^2^	During treatment	3.59 ± 1.38 b	1.26 ± 1.07 a
Apple surfaces	CPD	Pre-treatment	2.02 ± 0.17 a	2.00 ± 0.00 a
		Post-treatment	3.96 ± 1.18 b	2.08 ± 0.36 a
	FD	Pre-treatment	2.08 ± 0.28 a	2.01 ± 0.08 a
		Post-treatment	2.40 ± 0.23 a	2.00 ± 0.08 a

1 The detection method had a detection limit of 0 log_10_ MPN/100 mL of fungicide solution or 2 log_10_ CFU/apple.

2 CPD and FD indicate commercial packinghouse drencher and field drencher, respectively.

3 Average concentrations ± standard deviations in log_10_ MPN /100 mL and log_10_ CFU /100 mL for solutions and fruit surface, respectively. Data are the mean of 96 values from 12 apples and 8 replicate lots per treatment.

4 Values within the same column followed by different letters were significantly different by Tukey’s test at *p* ≤ 0.05.

## 4 Discussion

The newly developed FD was optimized for spray coverage and carries approximately five times less fungicide solution than the traditional CPD. As used in this study, the FD applies approximately 5.6 L of the fungicide solutions per bin, 50% less than the estimated 12 L through the CPD. Despite this difference, deposition patterns were equal or better through the FD likely because bins are not stacked and that 90% of the fungicide solution is retained on the fruits and bins during FD drenching. Comparatively, the concentration of the active ingredient strongly correlated (R^2^ > 0.83) with the number of bins drenched via the CPD and fungicide (a.i.) loss was estimated to be 40, 36 and 35% for TBZ, FDL and PYR, respectively, between 0 and 600 bins. Apples treated through the CPD were collected after approximately 200 bins had been treated, and it is possible that residue levels may be lower on CPD-treated fruit at the end of the lifespan of the tank. As expected from the “shower-down” nozzle, more deposition on the top of bins occurred on some occasions, but it was not always significantly different from the middle and bottom of the bins.

Spray coverage results were further supported by the fungicide residue levels detected on apples. Thus, FDL and TBZ levels were not significantly different between bin sections in 2021, whereas apples at the bottom received less PYR in 2022. Residue levels of FDL, TBZ, and PYR were all below the maximum residue levels of 5, 10, and 15 mg/kg, respectively,^1^ for both FD and CPD. The lower fungicide residue levels at the bottom of the bins treated through the FD are unlikely to reduce their efficacy as the minimum residue levels required for appropriate control are met for all three fungicides (Amiri, personal communication). Using a different bin system (0.38 m × 0.38 m × 0.91 m), Rosenberger et al. (2010) estimated that only 40% of fruit received adequate coverage at the bottom of the bins. However, the residue levels were not assessed and volume/bin used in their study was only 500 mL, significantly lower than the 5.6 L applied through the FD. Other studies reported that the method of fungicide application impacts the residue levels on fruit and that high-volume drencher usually results in higher residue levels (Kanetis et al., 2008; Kellerman et al., 2014, 2018). The FD is practical as it can be used to treat fruit immediately after harvest at the vicinity of orchards and therefore may protect fruit from infections that start on fresh wounds caused during harvest, transportation and handling at the storage facility (Amiri and Bompeix, 2005a). The FD is a portable system that can be transported between orchards but can also be used at vicinity of packinghouses. The spray turnout is only slightly higher through the CPD, which treats approximately 192 bins/h, when three bins are stacked, versus approximately 160 unstacked bins/h for the FD. Besides the mentioned benefits, future economic analyses and risk analysis accounting for changes in labor, waste management costs, decay, and food safety management are needed to accurately assess the economic benefits of the FD. It is a complex analysis in that operation of the FD requires more operational hours to move bins from the orchards to the drencher then to a semi-truck, however, the risk of potential introduction of fungal and food-borne pathogens must be assessed in the return on investment.

While the FD and CPD drenchers reduced the total microbiota on the apple surface after drenching except in one CPD-treated lot (Hc1139) in 2022, the risk that spores of pathogens known to cause decay in storage spread through drenching may be season- or fungicide-dependent. The higher overall fungal density observed on the apple surface in 2022 is most likely caused by the wetter conditions which occurred during the 2022 growing season compared to 2021. Furthermore, at the time point when apples were sampled from the CPD in 2022, the density of fungi in the PYR tank solution was about 27 CFU/mL compared to 100 CFU/mL after 600 bins, it is therefore expected that CPD-drenched apples would carry a higher spore density at the end of the tank lifespan. Sanderson and Spotts (1996) reported 12 *Penicillium* spp. in three TBZ CPD-drencher types from the PNW with densities ranging from 83.3 to 1,675 CFU/mL which is significantly higher than densities found in this study. No indication was provided regarding the number of bins drenched at the time of sampling, but packers used to recycle fungicides solutions for up to 2,000 bins (Spotts and Cervantes, 1993) which is not practiced anymore.

The two major postharvest pathogens known to spread through water recirculation are *Mucor* and *Penicillium* spp., the causal agents of blue mold and mucor rot, respectively. *Mucor* spp. was not isolated from the surface of apples treated with either drencher in this study, and the frequency of *Penicillium* spp. on fruit was relatively low at harvest confirming that infection by this pathogen occur mainly after harvest (Spotts et al., 1988; Amiri and Bompeix, 2005b). However, there was evidence of increased fruit contamination with *Penicillium* spp. spores via the recycling CPD in 2022 as their density increased 7.5 to 15-fold compared to the control in three Honeycrisp lots (Hc1139, Hc1156, and Hc11918). Meanwhile, there were significantly less spores of *Penicillium* spp. on apples of 75% of lots drenched with the FD compared to the CPD and the control. This may indicate that the combination of TBZ and FDL in 2021 had a better efficacy against *Penicillium* spp. that may be resistant to either fungicide or that spores that are PYR-resistant have accumulated in the CPD at the time the apples were drenched in 2022. The most frequently isolated culturable fungus was *Alternaria* spp., a ubiquitous carpoplane colonizer and a secondary postharvest pathogen of pome fruits (Texidó et al., 1999; Volschenk et al., 2016; Amiri and Ali, 2016). The FD and CPD equally reduced the carpoplane population of *Alternaria* spp. on apples treated with TBZ + FDL in 2021, whereas in 2022, *Alternaria* spp. increased in three and two lots on FD- and CPD-treated apples, respectively, post-drenching. Since spores of *Alternaria* spp. originate from the orchard, it is unlikely that spores were spread though the FD tank solution but rather due to different apples within the bins carrying different spore loads. Moreover, the large volume applied through the CPD may detach more spores from the apple surface than the FD. Similar seasonal variations between lots were reported on pears post-drenching via a recycling drencher (Volschenk et al., 2016). Despite some discrepancies, the FD is more likely to limit the spread of spores better than the CPD especially under higher disease pressure.

After 8 months of storage in RA and ~ 78% relative humidity (RH), the FD provided a greater efficacy in reducing the overall disease incidence in 50% of apple lots compared to the CPD, whereas equal efficacy was seen in the other two Honeycrisp lots, a cultivar highly susceptible to postharvest diseases. The efficacy of the FD in mitigating postharvest diseases was particularly evident in 2022, when the disease pressure was higher, as significant reductions were observed compared to the CPD in all but one lot. In the larger commercial trial including Honeycrisps and Gala apples from seven lots stored in CA at RH > 90%, the overall disease incidence was lower in 57% of the lots treated with PYR through the FD, albeit not always significantly compared to the CPD. The most prevalent postharvest diseases encountered in 2021 and 2022 were blue mold and gray mold. While the incidence of gray mold was either equivalent between the FD and the CPD or significantly lower in fruit treated through the CPD, the incidence of blue mold was significantly reduced by the non-recycling FD in 75% of the lots treated in 2021 and 2022. In the controlled atmosphere (CA) commercial trial, the incidences of blue mold and Mucor rot were reduced in 71% of Honeycrisp and Gala lots treated through the FD at harvest. Like in RA conditions, the incidences of gray mold, Alternaria rot, and bull’s eye rot, caused by the preharvest pathogens *Botrytis* spp., *Alternaria* spp., and *Neofabraea* spp., respectively, were higher in 50% of the fruit lots treated through the FD compared to the CPD after 10 months in *CA.*

Previous studies comparing recycling and non-recycling drenchers, with different designs than our, found the recycling drenchers to be more effective in reducing blue mold on inoculated Cortland and Empire apples (Rosenberger et al., 2010), and hypothesized that the non-recycling drencher would be less effective against field pathogens like *Botrytis* spp. (Rosenberger, 2011). Variable efficacy of recycling drenchers versus other methods have been reported in the citrus green mold fungus (Kanetis et al., 2008; Kellerman et al., 2014, 2018). The present study considered the overall disease incidence in whole bins, not by section, but more decayed fruits were observed at the bottom of the bins. Although the residue levels were above the minimum levels (0.5 to 2 mg/mL) needed to control sensitive isolates of the above pathogens, it is plausible that the relatively lower levels observed in the middle and lower sections of the bins treated through the FD may have not provided the anticipated efficacy against some of these preharvest pathogens, as opposed to a higher efficacy observed against wound pathogens like *Penicillium* spp. and *Mucor* spp. Furthermore, all lots used in this study were not treated with preharvest fungicides, which may be highly recommended to further enhance the efficacy of the FD against the preharvest pathogens. The FD has already been used by packers in the PNW in the past three years and feedback was positive in terms of reducing postharvest losses. Additional commercial trials testing different cultivars and fungicides are necessary to verify these observations, which may warrant additional adjustments in the volume of fungicide applied and the nozzle types utilized in the FD.

Cross-contamination with foodborne pathogens from postharvest water is recognized as a large food safety risk for fresh produce if water is not properly used and maintained (Possas and Pérez-Rodríguez, 2023; Carstens et al., 2019; Murray et al., 2017). Lab research looking at cross-contamination risk of apples found that washing apples inoculated with ~6.5 log CFU of *Listeria monocytogenes*/apple for 2 min in water transferred ~4.6 log CFU/mL and ~ 4.0 log CFU/apple of *L. monocytogenes* to the wash solution and non-inoculated apples, respectively (Sheng et al., 2020). Generic *E. coli* has been widely used as a water quality indicator to ascertain the likelihood that a water source is under the influence of fecal contamination. However, it is not a direct reflection of fecal contamination as studies have shown that growth of generic *E. coli* in the environment is possible, confounding its utility as a fecal indicator (Nowicki et al., 2021). Likewise, generic *E. coli* and the broader coliform group to which they belong, have been widely used to assess the transfer risk of bacterial foodborne pathogens, such as *Salmonella* and Shiga-toxigenic *E. coli* in field studies (Clarke et al., 2017). Therefore, differences observed in populations of *E. coli* and total coliforms on apples before and after FD and CPD drenching suggest that the CPD has a greater risk of cross-contamination compared to the FD, similar to the risk of spreading spore of plant pathogens. While pathogenic strains could not be employed in the present study, results support the notion that the FD reduces the risk of cross-contamination, including from foodborne pathogens, thereby enhancing overall food safety. Further evaluating the cross-contamination risk by modeling the transfer of inoculated surrogate organisms with phenotypic markers in both systems would be beneficial to help inform risk assessments tied to food safety.

Additionally, postharvest water that comes into contact with crops must have no detectable *E. coli*/100 mL based upon water quality criterion in the U.S. Produce Safety Rule (PSR; 21 CFR Part 112). While both drenchers had populations of *E. coli* recovered that are contributed from fruit and bins, the FD fungicide solution is not recirculated, contrary of the CPD water, which is recycled until it reaches the end of life based on the number of bins treated. With a population of 3.03 ± 1.36 MPN/100 mL *E. coli* recovered in CPD fungicide solutions; it is obvious that water will not meet the water quality criteria specified in the PSR as the indicator concentration increases with each subsequent pass through the system. These findings have significant implications for regulatory compliance, as continued use of recirculated fungicide solutions in CPD systems could lead to failure to meet the PSR’s microbial water quality standards. Since total coliform and generic *E. coli* accumulated in both drenchers, the addition of antimicrobial agents compatible with fungicides without affecting their efficacy, could effectively reduce bacterial levels in both systems. PAA as used in this study may have further reduced total coliform and generic *E. coli* better in the FD. Future work to optimize sanitizer use and examine compatibility with fungicides within a single use and recirculated drencher is needed.

## Data Availability

The raw data supporting the conclusions of this article will be made available by the authors, without undue reservation.

## Footnotes

^1^ https://nwhort.org
