# Supplementary figures and images for: Efficiency of a non-recycling postharvest fungicide drencher to enhance management of apple decay and food safety
Source: Front Microbiol. 2024 Dec 19;15:1509368. doi: 10.3389/fmicb.2024.1509368 (PMC11693708; doi:10.3389/fmicb.2024.1509368)

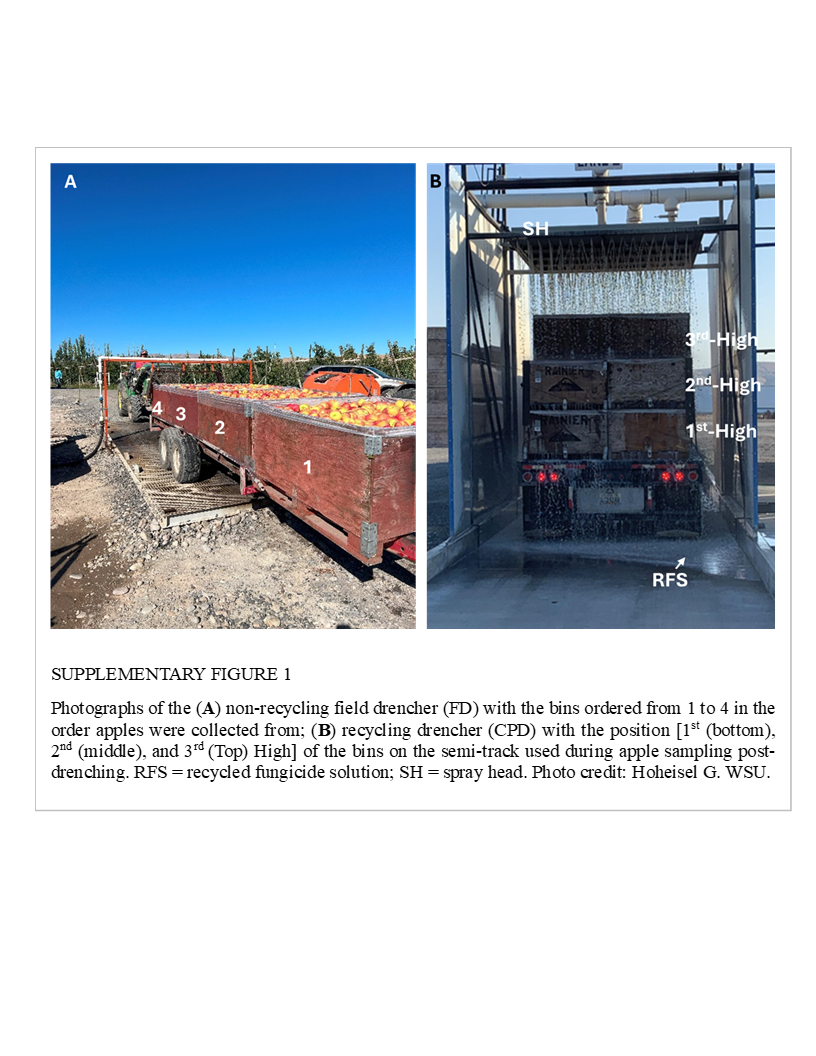

Supplement: Supplementary file 1 [file Image_1.tif]
